# Leveraging CT-based online adaptive radiotherapy for dose escalation in hypofractionated radiotherapy for locally advanced unresectable lung cancer

**DOI:** 10.3389/fonc.2026.1777077

**Published:** 2026-05-07

**Authors:** Simran Alexandria Polce, Nour Nasser, Celina Chiodo, Janelle Brewer, Sarah Dooley, Ibrahim M. Oraiqat, Jacqueline M. Andreozzi, Vladimir Semenenko, Gage Redler, Stephen A. Rosenberg

**Affiliations:** Department of Radiation Oncology, H. Lee Moffitt Cancer Center & Research Institute, Tampa, FL, United States

**Keywords:** adaptive radiotherapy, dose escalation, locally advanced lung cancer, NSCLC, unresectable lung cancer

## Abstract

Standard treatment for locally advanced (LA) lung cancer is surgery or concurrent chemoradiotherapy (CRT), but many patients are ineligible due to comorbidities. Sequential radiation (RT) and chemotherapy offer lower local control (LC) rates. Dose escalation and hypofractionation are emerging options for locally advanced (LA) NSCLC patients ineligible for surgery or chemoradiotherapy. However, toxicity rates remain unacceptable (G3-esophagitis up to 24%, G3-pneumonitis up to 12%). We have developed a novel approach that leverages CT-based online adaptive radiotherapy (oART) to deliver a simultaneously integrated boost while respecting OAR constraints. The oART workflow consists of image acquisition, patient modeling/contouring, plan generation/selection, verification image match (CBCT-to-CBCT), and treatment delivery. Targets were rigidly propagated and aligned daily; critical OARs were recontoured to account for inter-fraction variability and ensure OAR tolerances were not exceeded for daily treatment. Here, we outline our technical approach.

## 1 Introduction

Lung cancer remains the third most common cancer diagnosis after breast and prostate cancers in the United States (1). Nearly one-third of NSCLC patients are classified as having locally advanced cancer at diagnosis (2). Locally advanced lung cancer encapsulates patients with Stage IIB (T3N0) and Stage III disease (3). The mainstay of treatment for patients deemed surgical candidates is a lobectomy with mediastinal lymphadenectomy with either neoadjuvant or adjuvant systemic therapy. This generally requires disease confined to a single lobe and single ipsilateral nodal station. Once patients have multilevel N2 or N3 disease or have bulky mediastinal nodes, concurrent chemoradiotherapy becomes the treatment of choice (4). However, patients are often deemed ineligible for concurrent chemoradiotherapy for similar reasons to surgery (poor KPS, poor respiratory function, etc.). As a result, patients often receive sequential radiotherapy and chemotherapy (5, 6). This approach notably sacrifices local and distant control. Radiotherapy regimens such as 4,500 cGy/15 Fx, and 3,000 cGy/10 Fx have been proposed as alternatives to 6,000 cGy/30 Fx but are palliative in nature with a lower biologically effective dose (BED_10_). There is growing interest in dose escalation to improve local control. One such popular regimen is 6,000 cGy delivered in 15 fractions (Fx) (7). While found to be tolerable, dose escalation regimens tend to result in high rates of toxicity (8) (pneumonitis and esophagitis) and had poor OS likely secondary to poor functional status leading to limited data on long-term toxicity (7, 9). This is largely attributed to the nature of having a central target (primary mass, mediastinal lymph nodes) adjacent to mobile critical organs at risk (OARs).

In this paper, we seek to outline a novel approach to dose escalation in unresectable locally advanced lung cancer using the Ethos [version 1.1, Varian, a Siemens Healthineers Company, Palo Alto, CA (RRID: SCR_017372)] Cone-Beam Computed Tomography (CBCT)-guided ring-gantry online adaptive radiotherapy (oART) system. The Ethos system operates on the Halcyon treatment machine (Varian, a Siemens Healthineers Company, Palo Alto, CA) with capabilities of fast gantry rotation (4 revolution per minute (RPM) when treating and 6RPM when imaging) facilitated by the closed-bore design. It has a single 6-MV flattening filter free (FFF) beam collimated by double-stacked and staggered multi-leaf collimators (MLCs) providing an effective leaf width of 0.5 cm at the isocenter over a 28 × 28 cm^2^ field (10). Ethos uses the Acuros XB dose calculation algorithm, a linear Boltzmann transport equation solver. During the online adaptive workflow, the system utilizes deformable image registration, from the initial CT to the daily CBCT, to provide voxel-by-voxel mapping of material and density information for daily dose calculation. This process represents a more sophisticated process than simple bulk density assignment to contours, which is often used in MR-only planning approaches (11). Using artificial intelligence (AI) and/or deformable image registration, a subset of site-specific normal structures (“influencers”) are automatically contoured, followed by targets and remaining OAR generation. The user may optionally edit these contours further or simply accept them as they are. Further structure derivations, previously defined in initial planning, are then applied (e.g., margin expansions from gross target volume (GTV) to planning target volume (PTV), daily anatomy based high-dose regions a certain distance from critical OARs). The system then generates the scheduled (initial plan recalculated/evaluated on the new anatomy) and adapted (initial plan reoptimized on the new anatomy) plans, both maintaining the original beam geometry (gantry and collimator angles) used in the initial plan. Detailed information regarding Ethos treatment management system has been previously presented (12). For real-time patient monitoring, the system was integrated with the AlignRT InBore (Vision RT, London, UK) surface guidance system to optimize treatment delivery.

## 2 Patient selection

Hypofractionated radiation requires careful patient selection. Specifically, patients must not be eligible for definitive surgical intervention or concurrent chemoradiotherapy. We prioritize offering this approach to patients with large or centrally located tumors, patients with disease exhibiting large respiratory motion/variability, patients at risk of atelectasis or obstructive pneumonitis, and/or patients with multiple central targets. In this approach, patients are selected based on these clinical and anatomical criteria. In addition, careful consideration is given to select patients who are able to tolerate prolonged treatment time given the nature of the adaptive workflow. Generally, patients are required to lay flat and keep their arms extended overhead for 30–45 minutes.

## 3 Simulation

Simulation for an adaptive workflow is similar to the traditional lung radiotherapy approaches. A patient is brought to the CT simulator and placed in the supine position with their arms overhead. Both arms above the head is ideal for maximizing feasible beam angles; however, patients with limited mobility may be simulated with one arm up (ipsilateral to tumor) or both arms down if absolutely necessary. In this scenario, immobilization should secure arms down to ensure consistent placement and certain beam geometry may need to be utilized to best avoid entrance in the arm region. A Long BodyFix (Elekta, Stockholm, Sweden) is used for patient immobilization. A 4D-CT (no more than 3-mm slice thickness) is then obtained to account for respiratory motion by capturing the entire respiratory cycle via breathing trace amplitude-based binning of acquired projection data for reconstruction of 10 separate 3D images representing 10 separate phases of the breathing cycle. An average CT is then reconstructed from the 4D-CT data to be used as the primary CT for dose calculation purposes. The average CT is generated by integrating raw projection data for reconstruction of a single 3D image utilizing projections acquired from all breathing phases. A free-breathing, contrast-enhanced (arterial phase) CT is obtained to help with the delineation of lymph nodes and vasculature in the mediastinum, if medically feasible (i.e., no iodine contrast allergy and sufficient renal function). The patient setup isocenter is then selected by the treating physician (e.g., approximate centroid of target(s), carina), and external marks are placed on the patient.

## 4 Contouring

The 4D-CT is used for target delineation by the physician. Obtaining a free-breathing contrast-enhanced arterial-phase CT is highly recommended at the time of simulation for target and OAR delineation. If available, a PET-CT with rigid fusion should be utilized. The target is contoured across all phases of the respiratory cycle to generate the internal gross tumor volume (IGTV_4500_) structure. Similarly, all involved nodes should be contoured across the respiratory cycle to delineate a nodal IGTV_4500_. In cases of primary lung carcinomas with definitive treatment intent, a 7-mm isotropic expansion of the IGTV_4500_ structure is used to generate the internal target volume (ITV_4500_) for the primary and nodal structures with the goal of treating microscopic disease (ITV_4500_ is the equivalent of an ICTV at other institutions). In these cases, ITV_4500_ is manually edited to respect natural barriers to spread such as the chest wall, bone, esophagus, and great vessels, unless there is concern for direct extension or invasion by the primary disease. In cases where patients have metastatic disease (Stage IV), we currently do not utilize an ITV (which is our institutional language for microscopic coverage also known as ICTV at other institutions). In these cases, an ITV is omitted as the primary purpose of treatment is to target a central target to reduce disease burden or to address symptoms. The elimination of the ITV is in line with the overall goal of limiting patient toxicity given that these patients are incurable. In all other cases, an ITV is recommended as the intent of treatment is curative and an ITV is in line with current curative locally advanced contouring guidelines. A 5-mm radial and 7-mm superior/inferior expansion is then made off the edited ITV_4500_ to create the planning target volume (PTV); if no ITV_4500_ is made, then a 5-mm radial and 7-mm superior/inferior expansion is made off the IGTV_4500_ to create the PTV. IGTV_4500_ and ITV_4500_ will often be contoured separately for primary and nodal targets before combining/expanding to create the PTV in order to allow evaluation of dosimetric characteristics for individual targets as well as facilitate separate rigid alignment to daily CBCT accounting for potential independent changes in target positions.

Critical OARs are best contoured on the weighted average CT. It is important for the walls of the luminal structures to be appropriately included; otherwise, this may lead to incidental hot spots within the walls during initial or adaptative planning. Mandatory OARs include the heart, lungs, proximal bronchial tree (PBT), esophagus, spinal cord, great vessels, skin (defined as 5-mm-thick rind of the patient body habitus) and chest wall. Optional OARs, depending on location of the tumor, include ipsilateral brachial plexus, thyroid, stomach, and liver. OARs should be contoured according to the NRG/RTOG Organs at Risk in Thoracic Radiation Therapy Atlas.

The PBT and esophagus are expanded by 3 mm isotopically to create OAR_PRV structures utilized in the planning process for optimization of dose escalation. An OAR_eval structure is generated by taking the PTV and expanding 2 cm sup/inf and 3 cm radially. The OAR_eval structure is extrapolated from ultracentral dose escalation data previously published (13). Daily recontouring during the adaptive process will focus on OARs within this structure as this represents a region containing potentially relevant doses with respect to OAR tolerances and the explicit focus helps to streamline contouring during online adaptation.

## 5 Planning

**Figure 1:**
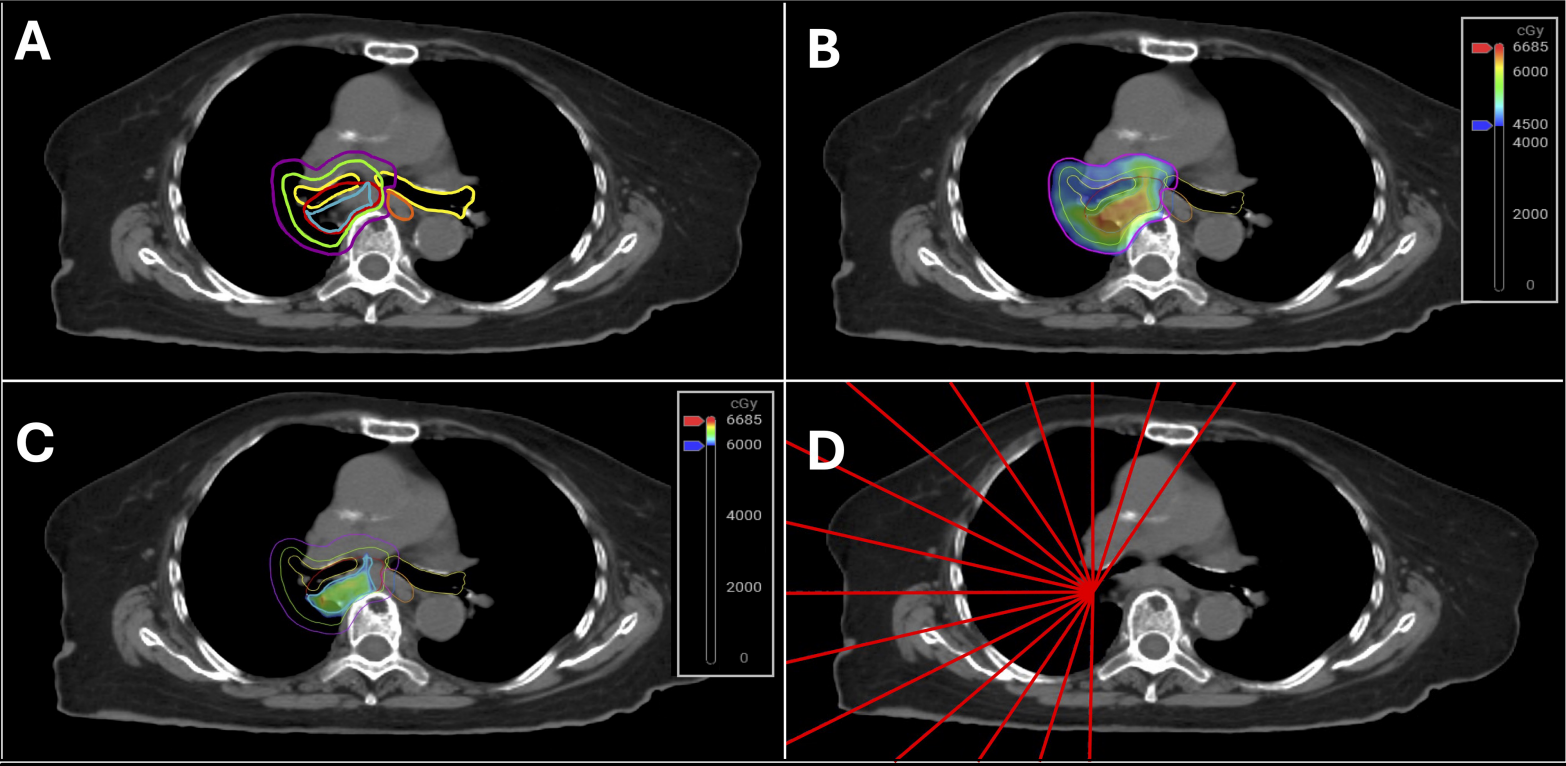
CT axial view with pre-set soft tissue window/level. **(A)** Demonstrated are the critical OARs–the PBT (yellow) and Esophagus (orange). Additionally, the IGTV_4500 (red), ITV_4500 (green) and the PTV_4500 (purple). The IGTV_6000 (cyan) is derived from the PTV_4500 minus the OAR_PRV (3mm expansions of OARs). **(B)** demonstrates the 4500cGy prescription dose, **(C)** demonstrates the dose escalation volume, and **(D)** demonstrates an example beam geometry.

Our novel approach is to treat the entire target (PTV_4500_ and PTV_4500_LN_) to 4,500 cGy in 15 fractions (300 cGy/Fx) while incorporating a simultaneous integrated boost (SIB) optimization target to safely escalate as much of the primary tumor to 6,000 cGy (400 cGy/Fx) as possible (>90% covered by 6,000 cGy). This IGV_6000_ structure is derived by subtracting critical structures plus a margin expansion (OAR_PRV) from the IGTV_4500_ [i.e., IGTV_6000_ = IGTV_4500_ – OAR_PRV, where OAR_PRV = (esophagus + 3 mm) + (PBT + 3 mm)] (**Figures 1A–C**). To ensure appropriate PTV_4500_ target coverage without violation of OAR constraints, more practical evaluation structures were created in case the PTV_4500_ intersects with critical structures (e.g., brachial plexus, esophagus). For example, the structure eval_PTV_4500_ = PTV_4500_ – esophagus is used to achieve optimal target coverage (>95% of this structure covered by 4,500 cGy) while still avoiding the 4,500 cGy circumferential esophagus dose. Additional optimization structures were created to crop PTV_4500_ or PTV_4500_LN_ away from the esophagus [e.g., (opti_PTV_4500_ = PTV_4500_ – (esophagus + 3mm)]. Our goal is to dose-escalate while respecting OAR dose constraints and target coverage goals, which are summarized in detail in **Tables 1**, **2**, respectively. The ideal application of these constraints would theoretically provide an isotoxic treatment, but this will require eventual evaluation of patient outcomes in future work. In supplemental **Table 3**, we have provided the fractional dose constraints and the application of these per fraction constraints on a representative patient who had several large mediastinal lymph nodes in level 4. As demonstrated in **Table 3**, there are significant differences in the OAR doses abutting the target volume. Ultimately, the adapted plan was chosen due to the ability to decrease dose to the OARs abutting the target. Hypofractionated lung radiotherapy lacks a central consensus on constraints. Our constraints are extrapolated from previous non-adaptative dose escalation studies (7, 14, 15). All treatment plans were created within the Ethos treatment management system. The arrangements for treatment plans in this work utilized 15–20 custom beams with fixed-field intensity-modulated radiation therapy (IMRT). These were generally placed such that beam entrances shared laterality with the target (i.e., 330°-180° clockwise for left-sided or 30°-180° counter-clockwise for right-sided). An example of beam arrangement in depicted in **Figure 1D**. Volumetric modulated arc therapy (VMAT) has not been used for these treatments as the plan optimization times are appreciably longer than those of IMRT and therefore can extend patient time on the table and increase treatment uncertainty (12, 16, 17).

**Table 1:** Planning objectives for critical OARs.

OAR objectives		
Organ at risk	Max dose	Dose-volume limit
Spinal cord	D_0.03cc_ < 3500 cGy	
SpinalCord_PRV05	D_0.10cc_ < 3800 cGy	
Lungs-ITV_4500_		D_mean_ < 1800 cGy V_500cGy_ < 65 % V_1800cGy_ < 37 % V_2000cGy_ < 30 % V_1550cGy_ < 1500 cc V_1630cGy_ < 1000 cc
Esophagus	D_0.03cc_ < 5130 cGy	V_4800cGy_ < 5 cc D_mean_ < 3000 cGy
Heart	D_0.10cc_ < 6300 cGy	V_3950cGy_ < 15 cc D_mean_ < 1000 cGy
Proximal bronchial tree	D_0.03cc_ < 5530 cGy	V_5130cGy_ < 5 cc
Great vessels: aorta, vena cava, pulmonary vessels	D_0.10cc_ < 6300 cGy	
Skin (5 mm)	D_0.03cc_ < 5540 cGy	V_4900cGy_ < 10 cc
Brachial plexus	D_0.03cc_ < 5060 cGy	D_0.10cc_ < 4500 cGy

Dose constraints are derived from previously published hypofractionated papers. Of note, there should be no circumferential prescription (4,500 cGy) dose to the esophagus. Spinal cord PRV uses a 5-mm margin. Daily evaluation will be based on the per-fraction equivalent (i.e., cumulative dose goal divided by 15).

**Table 2:** Planning objectives for dose coverage of the primary targets as well as the derived simultaneous integrated boost target.

Target volumes	Min dose [cGy]	Dose-volume goal
IGTV_4500_	4500	
ITV_4500_	4500	
PTV_4500_*		V_4500cGy_ > 95%
IGTV_6000_	5700 *(4500)*	V_6000cGy_ ≥ 95% *(90%)*

Acceptable variation in italicized parenthesis. Note, min doses represent minimum dose to the structure excluding the 0.03cc receiving the lowest dose (i.e., D_min,0.03cc_). *This may be substituted with edit_PTV_4500_ in cases where overlap with esophagus compromises PTV_4500_ coverage to avoid circumferential esophagus dose. Daily evaluation will be based on the per-fraction equivalent (i.e., cumulative dose goal divided by 15).

**Table 3:** Example dosimetric results for a single representative online adaptive fraction using the approach presented in this work.

OAR objectives			Achieved plan metrics	
Organ at risk	Max dose per fraction	Dose-volume limit per fraction	Scheduled plan	Adapted plan
Spinal cord	D_0.03cc_ < 187 cGy		D_0.03cc_=174 cGy	D_0.03cc_=177 cGy
SpinalCord_PRV05	D_0.10cc_ < 253 cGy		D_0.10cc_ =204 cGy	D_0.10cc_ =208 cGy
Lungs-ITV_4500_		D_mean_ < 87 cGy V_33cGy_ < 65 % V_120cGy_ < 37 % V_133cGy_ < 30 % V_103cGy_ < 1500 cc V_109cGy_ < 1000 cc	D_mean_ = 79 cGy V_33cGy_ = 60.1 % V_120cGy_ =26.1 % V_133cGy_ =21.5 % V_103cGy_ =640.14 cc V_109cGy_ =607.33 cc	D_mean_ = 77 cGy V_33cGy_ =59 % V_120cGy_ = 24.7% V_133cGy_ = 20.4 % V_103cGy_ = 618.43cc V_109cGy_ = 580.24 cc
Esophagus	D_0.03cc_ < 342 cGy	V_320cGy_ < 5 cc D_mean_ < 200 cGy	D_0.03cc_ =382 cGy V_320cGy_ =1.25 cc D_mean_ =127 cGy	D_0.03cc_ =330 cGy V_320cGy_ = 0.27 cc D_mean_ =123 cGy
Heart	D_0.10cc_ < 420 cGy	V_263cGy_ < 15 cc D_mean_ < 67 cGy	D_0.10cc_ = 376 cGy V_263cGy_ <=9.45 cc D_mean_ =47 cGy	D_0.10cc_ = 397 cGy V_263cGy_ = 9.93 cc D_mean_ =47 cGy
Proximal bronchial tree	D_0.03cc_ < 369 cGy	V_342cGy_<5.0 cc	D_0.03cc_ =416 cGy V_342cGy_= 3.57 cc	D_0.03cc_ =359 cGy V_342cGy_= 0.52 cc
Great vessels: aorta, vena cava, pulmonary vessels	D_0.10cc_ < 420 cGy		D_0.10cc_ =366 cGy	D_0.10cc_ = 388 cGy
Skin (5mm)	D_0.03cc_ < 369 cGy	V_327cGy <_10.0 cc	D_0.03cc_ =169 cGy V_327cGy_ =0 cc	D_0.03cc_ =165 cGy V_327cGy_ =0 cc
Brachial plexus	D_0.03cc_ < 337.3 cGy	D_0.10cc_ < 300 cGy		

This serves to illustrate the variables that may be considered in order to decide if the scheduled or adapted plan will provide superior dosimetry. In this instance, the adapted plan was selected for treatment.

While clinical goal prioritization for dose optimization is of course dependent upon specific patient disease/anatomy/clinical considerations, the general approach consisted of placing highest priority on maximal doses to critical OARs (esophagus, PBT, etc.), followed by target coverage. Beyond this, lung volume and other lower dose/volumetric goals for the critical OARs are optimized, followed by any geometric helper goals (e.g., further optimizing coverage to a small (3 mm) rind of the target that may be in lower density lung tissue or explicitly pushing dose off of the contralateral esophagus wall).

## 6 Online adaptive workflow

Our institution utilizes the following steps as part of the original planning and online adaptive workflow process. These steps are part of a formal checklist, and we encourage new users to develop and implement similar checklists to ensure the adaptive processes are followed in a consistent manner. Online adaptive radiotherapy requires coordination between teams of physicians, physicists, and radiation therapists. Given the nature of this 15-fraction treatment course, an attending radiation oncologist with adaptive experience is always present and leads the adaptative process on the first fraction. The attending physician also leads a treatment session at least once per week. Other treatment sessions are led by physicists with adaptive experience. For sessions not led by an attending physician, a daily offline check of the contours, treated plan, and verification image match (CBCT-to-CBCT) is performed. In addition, there is always an adaptive physician on site available to assist should an issue arise. This is felt to be a safe approach as targets are rigidly prorogated and not recontoured/adjusted unless by an attending physician (see workflow below).

### 6.1 Patient positioning and respiratory cycle definition

Daily adaptation is performed while the patient is positioned on the treatment table in the BodyFix in a position reproducing that at CT simulation. The patient is instructed to breathe naturally while an appropriate device is used to monitor the breathing cycle (e.g., optical surface guidance, spirometry, infrared markers). Care is taken to ensure that the surrogate monitoring breathing cycle provides a reasonable and reproducible representation of patient breathing. After confirmation of consistent breathing of the patient, a mid-breath CBCT is obtained with the patient in breath hold. This allows for the planning CBCT to capture the tumor/targets in a centralized position over the respiratory cycle and reduce motion artifacts (18). Once the daily planning CBCT is acquired, it is assessed to ensure adequate image quality for contouring/planning as well as sufficient field of view (FOV) to contain pertinent anatomy and appropriate tissue for dose calculation/evaluation. The CBCT imaging system provides a 70-cm-diameter FOV, which is generally sufficient to capture the full patient body habitus, particularly for centrally located targets. For laterally located targets or larger patients, where contralateral imaging may be incomplete, this loss of information has negligible dosimetric consequences as a unilateral beam arrangement is used for all treatments. This ensures that the anatomy outside of the FOV is not dosimetrically critical since it is outside of the primary treatment location and any relevant beam paths.

### 6.2 Target rigid propagation and re-contouring daily anatomy

**Figure 2:**
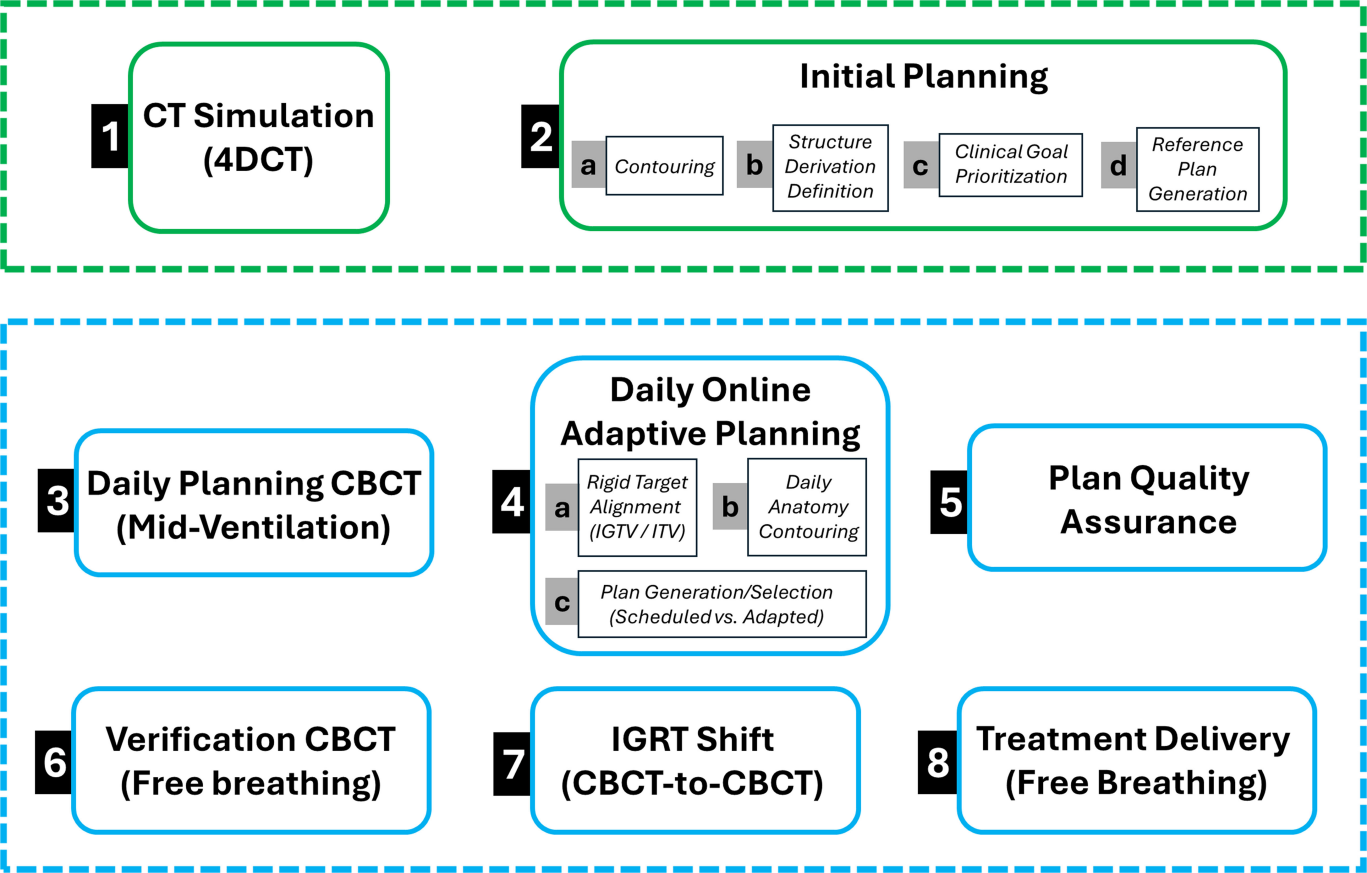
Depicted is the workflow model outlined in this paper. Steps 1 &2 comprise the initial planning and contouring process. Steps 3-8 the daily adaptative process which is repeated for each treatment fraction.

Following patient setup, CBCT acquisition, and AI-driven generation of relevant anatomical structures, attention is then given to the targets and remaining OARs. Within the Ethos workflow, there is no initial image match to align patients (the adapted plan isocenter is placed at the planning CBCT acquisition center, and the scheduled plan isocenter is determined via an automatically calculated rigid translational shift to match target centers). The IGTV_4500_, contoured on the initial 4DCT from CT Simulation to ensure breathing motion is adequately included, is rigidly aligned. This ensures that a consistent target region accounting for breathing motion is covered by the 4,500-cGy prescription dose daily (i.e., essentially providing an equivalent to standard non-adaptive IGRT treatment workflows relying on image matching for target alignment). This is followed by recontouring OARs based on daily anatomy. Only underived structures (e.g., IGTV_4500_, ITV_4500_, or OARs) are available for editing, serving as the basis for deriving PTVs, PRVs, and optimization/evaluation structures. To save time during the adaptive process, priority is given to the OARs within the OAR_eval structure as described above. Careful attention is given to ensure walls of luminal structures are included in volume delineation (19). Once contours are completed/verified by the adaptive physician or physicist, the new OAR_PRV structures of the day are created by a 3-mm isotopic expansion. The IGTV_6000_ is re-created by the preset Boolean function, which includes subtracting the OAR_PRV structures from the PTV_4500_ (**Figures 1A–C**). As a result of OAR variability in position, shape, and fill, the IGTV_6000_ volume inherently changes daily. During this process, the initial planning scan, contours, and plan as well as the first fraction planning CBCT, contours, and plan are available on an adjacent workstation for reference. An overview of the workflow is outlined in **Figure 2**. Steps 1 and 2 are part of the initial planning process; steps 3–9 are repeated during each fraction. **Supplementary Figure 4** exemplifies the interfractional variability of OARs despite utilizing a 4D scan during initial simulation. As highlighted in this figure, the esophageal contour created on the 4D scan still failed to encapsulate the variability of the esophagus.

### 6.3 Dose prescription and plan evaluation

**Figure 3:**
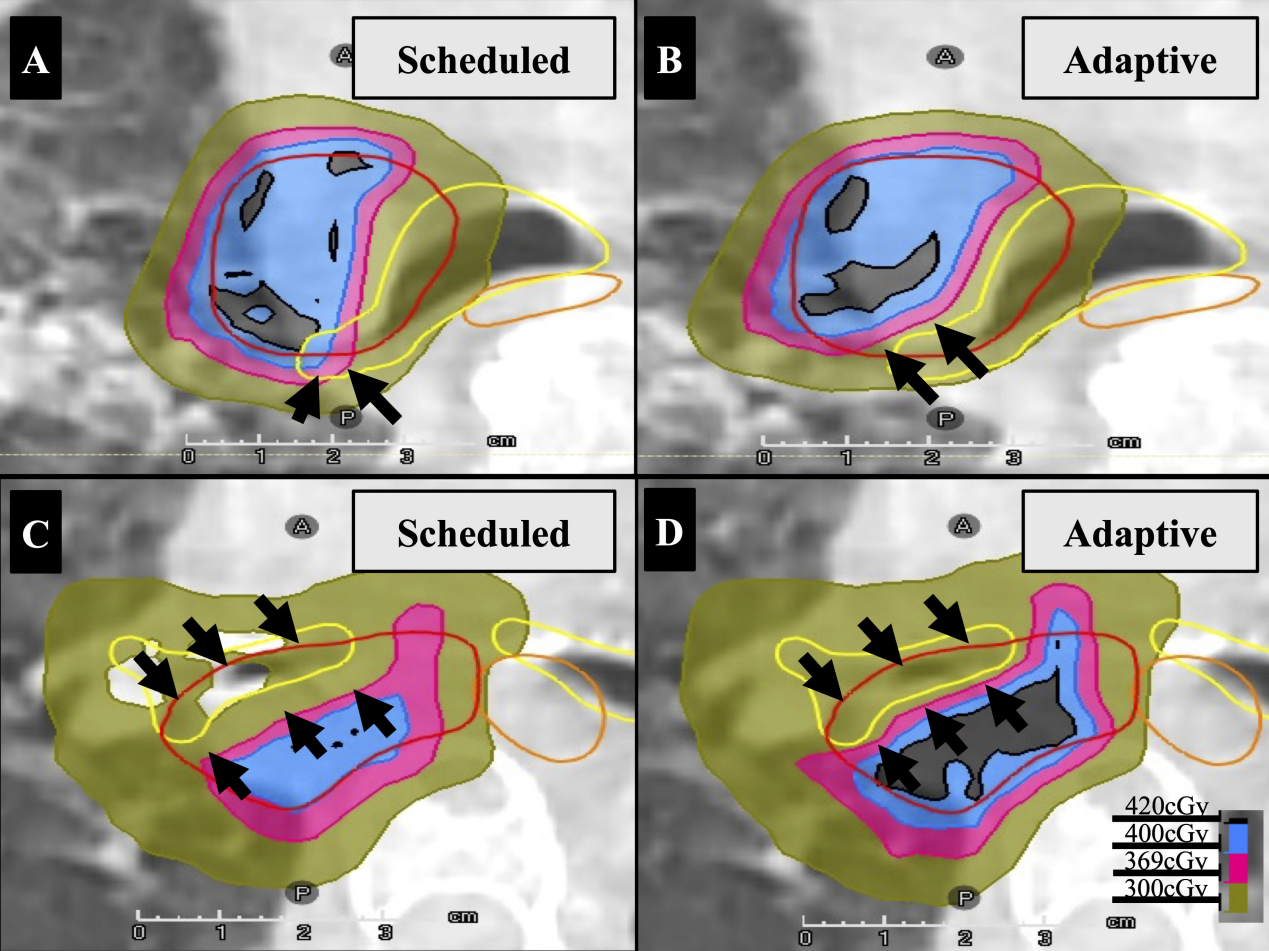
CT Axial view with pre-set lung window/level. **(A, B)** show improved PBT (yellow) sparing by critical dose in a single fraction. **(C, D)** illustrate enhanced IGTV (red) coverage in a single representative fraction. The PBT is yellow, and the esophagus is orange. Dose colorwash is scaled per fraction 420cGy=105% Rx (400cGy), 300cGy=IGTV Dmin, and 369cGy is PBT Dmax.

Once the re-contoured structures are approved by the team lead of the day (physician or physicist), the online adaptive plan optimization and calculation processes start. The primary prescription is 4,500 cGy (300 cGy/Fx) to the original PTV. The original PTV remains unchanged throughout the treatment course as the target is rigidly aligned, providing an equivalent of standard, non-adaptive image-guided radiation therapy (IGRT) treatment at this dose level. The scheduled plan utilizes the original beam geometry and beam segments to recalculate the dose on the image/anatomy of the day while the adapted plan is generated using the original beam geometry but modified beam segments to achieve the prescribed dose and OAR sparing based on the new anatomy. Both the scheduled and adapted plans are then compared visually using an isodose map and/or dose volume histogram (DVH). Quantitatively, the plans are compared by the achieved values for coverage goals and OAR dose constraints. Visual evaluation of dose distribution remains good practice, as it allows for inspection of hotspot location and coverage conformality. A representative fraction showing improved OAR sparing is demonstrated in **Figures 3A, B**. An example of visual inspection demonstrating improved target coverage is presented in **Figures 3C, D**. The most dosimetrically favorable plan is then selected by the treating physician or covering physicist after plan evaluation.

### 6.4 Dose delivery

Following plan selection, a second free-breathing verification CBCT scan is acquired immediately prior to treatment delivery. Given the time interval (31 ± 7 min) between the initial mid-breath hold daily planning CBCT, this verification scan allows for confirmation that the tumor remained within the planned IGTV_4500_ and that critical OARs have remained consistent. Any necessary translational shifts to correct for patient or organ motion are made at this time by the radiation therapist. Image alignment and associated shifts are confirmed by the physician or physicist leading the oART treatment session. It should be noted that contouring/planning on the mid-breath hold CBCT generally will underestimate OAR motion (not an issue for targets since the IGTV(s)/ITV(s) incorporating motion are rigidly aligned, thus preserving volumes) and thereby introduce a source of uncertainty and potential inaccuracy. However, although not quantified, based on our experience in this work, this is a reasonable tradeoff compared with the uncertainty associated with contouring critical OARs on a free breathing CBCT with motion artifacts. The uncertainty introduced by contouring on the breath hold CBCT is ameliorated by performing final alignment on the free breathing verification CBCT, which ensures that the 4,500-cGy target(s) are still well aligned and adequately encompass breathing motion. Additionally, this allows us to ensure average match of OARs (e.g., PBT based on carina) by reviewing image-to-image match and daily planning contours overlayed on free breathing verification CBCT.

A calculation-based IMRT plan quality assurance check of the selected plan is then performed using the Mobius3D system (Varian, a Siemens Healthineers Company, Palo Alto) before treatment delivery. Mobius3D is an independent secondary verification tool which utilizes a collapsed cone convolution superposition dose calculation algorithm and performs gamma analysis to ensure dosimetric accuracy of the newly generated plan. Treatment is delivered while the patient is free breathing, as target motion is encapsulated by the IGTV_4500_.

### 6.5 Patient compliance considerations

There have been a few instances where patient symptomatology has resulted in prolonged treatment times. Based on our experience, we suggest the following techniques to minimize risk of treatment interruption. Patients with a prior history of obstructive respiratory disease should be medically optimized prior to initiation of radiotherapy by their primary care physician or pulmonologist. They should take any as needed or “PRN” prescribed inhalers within 30 min of treatment session. Additionally, supplemental nasal cannula oxygen at a rate of 2 L/min may be utilized. For patients with a baseline cough, an over-the-counter or prescription anti-tussive agents, such as benzonatate, can be considered. Patients with a history of GERD should start a prophylactic PPI (i.e., Protonix) prior to radiotherapy or fast for 3-4 h prior to treatment session.

## 7 Post-treatment monitoring

Patients should be monitored closely during treatment with weekly physician assessments and evaluated in the immediate posttreatment period for toxicity. Patients continue with routine imaging for lung cancer after treatment, including a CT Chest with intravenous contrast every 3 months for the first year after radiotherapy, and then every 6 months thereafter. Patients may be eligible for further therapy per multidisciplinary team.

## 8 Conclusion

CT-based oART to allow for simultaneous integrated boost in the context of locally advanced lung cancer is both feasible and, from our institutional experience, provides a personalized treatment that optimizes target dose escalation and normal tissue sparing. The technique presented in this work combines more traditional methodology (motion encompassing targets rigidly aligned daily) with more novel online adaptation of high doses based on daily patient anatomy.

## Data Availability

The raw data supporting the conclusions of this article will be made available by the authors, without undue reservation.
