# Supplementary figures and images for: Leveraging CT-based online adaptive radiotherapy for dose escalation in hypofractionated radiotherapy for locally advanced unresectable lung cancer
Source: Front Oncol. 2026 May 7;16:1777077. doi: 10.3389/fonc.2026.1777077 (PMC13189974; doi:10.3389/fonc.2026.1777077)

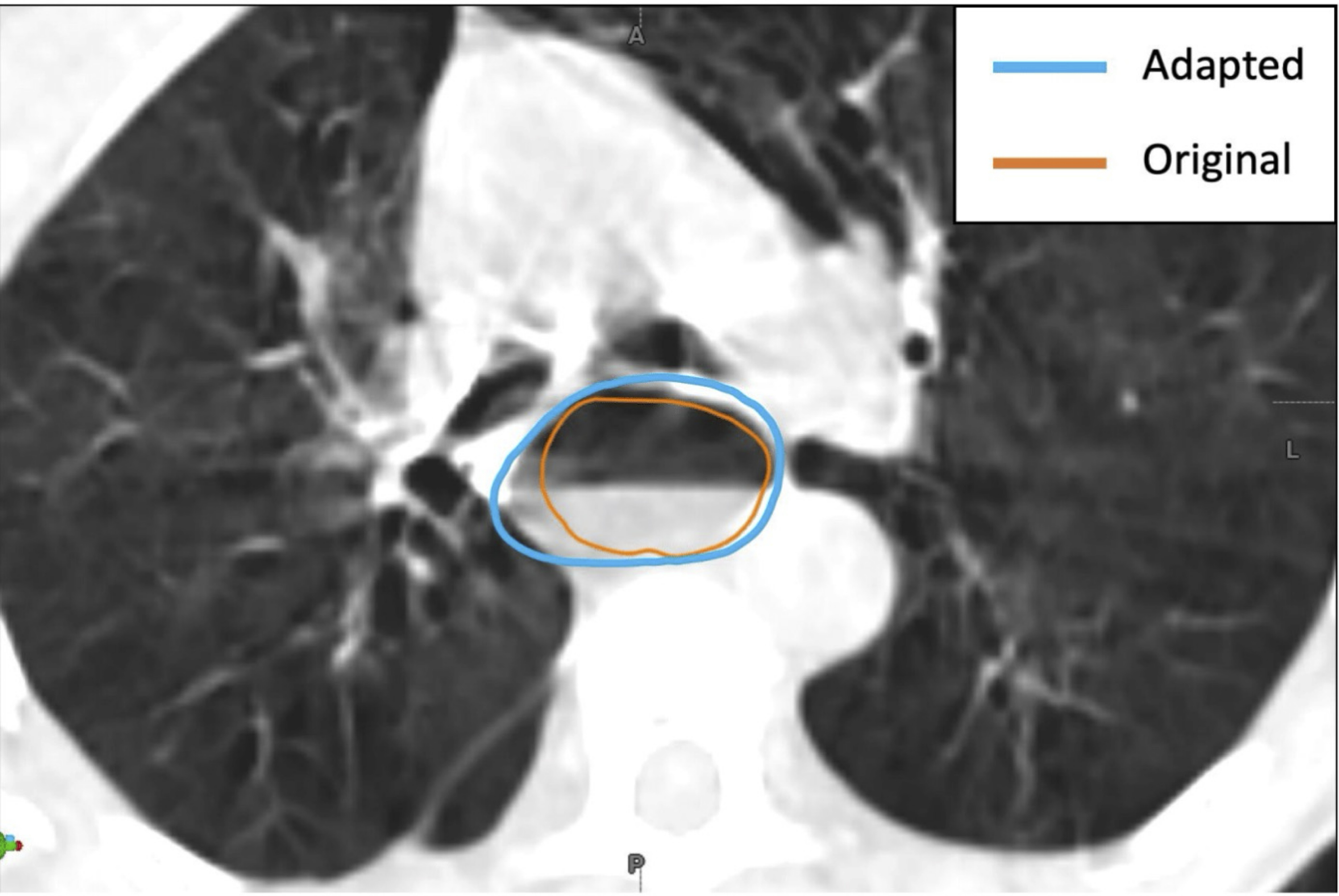

Supplement: Supplementary file 1 [file Image1.tiff]
